# Transfer of Knowledge on Pneumoconiosis Care Among Rural-Based Members of a Digital Community of Practice: Cross-Sectional Study

**DOI:** 10.2196/52414

**Published:** 2024-01-24

**Authors:** Brian Soller, Orrin Myers, Akshay Sood

**Affiliations:** 1 Department of Sociology, Anthropology, and Public Health University of Maryland Baltimore County Baltimore, MD United States; 2 Department of Family and Community Medicine University of New Mexico Health Sciences Center Albuquerque, NM United States; 3 Department of Internal Medicine University of New Mexico Health Sciences Center Albuquerque, NM United States; 4 Miners Colfax Medical Center Raton, NM United States

**Keywords:** community of practice, knowledge transfer, pneumoconiosis, telementoring, rural health care, transfer, information, rural, virtual community, lung diseases, lung disease, rural professionals, rural professional, multidisciplinary management, multidisciplinary, miners, miner, health equity

## Abstract

**Background:**

Given the re-emergence of coal workers’ pneumoconiosis in Appalachia and Mountain West United States, there is a tremendous need to train rural professionals in its multidisciplinary management. Since 2016, the Miners’ Wellness TeleECHO (Extension for Community Health Outcomes) Program held by the University of New Mexico, Albuquerque, and Miners’ Colfax Medical Center, Raton, New Mexico, provides structured longitudinal multidisciplinary telementoring to diverse professionals taking care of miners by creating a digital community of practice. Program sessions emphasize active learning through discussion, rather than didactic training. Professional stakeholder groups include respiratory therapists, home health professionals, benefits counselors, lawyers or attorneys, clinicians, and others. Rural-urban differences in knowledge transfer in such a community of practice, however, remain unknown.

**Objective:**

We aim to evaluate the role of the rurality of the patient or client base in the transfer of knowledge to professionals caring for miners using the digital community of practice approach.

**Methods:**

This is a cross-sectional study of 70 professionals participating in the Miners’ Wellness TeleECHO Program between 2018 and 2019. Drawing insights from social network analysis, we examined the association between the rurality of participants’ patient or client base and their self-reported receipt of knowledge. Our focal independent variable was the respondent’s self-reported percentage of patients or clients who reside in rural areas. We measured knowledge transfer sources by asking participants if they received knowledge regarding the care of miners during and outside of TeleECHO sessions from each of the other participants. Our dependent variables included the number of knowledge sources, number of cross-stakeholder knowledge sources, number of same stakeholder knowledge sources, and range and heterogeneity of knowledge sources.

**Results:**

Respondents, on average, identified 4.46 (SD 3.16) unique knowledge sources within the community, with a greater number of cross-stakeholder knowledge sources (2.80) than same stakeholder knowledge sources (1.72). The mean knowledge source range was 2.50 (SD 1.29), indicating that, on average, respondents received knowledge sources from roughly half of the 5 stakeholder groups. Finally, the mean heterogeneity of knowledge sources, which can range between 0 and 0.80, was near the midpoint of the scale at 0.44 (SD 0.30). Multivariable analyses revealed that as the rurality of patient or client bases increased, participants reported more knowledge sources overall, more knowledge sources from outside of their stakeholder groups, a higher knowledge source range, and greater heterogeneity of knowledge sources (*P*<.05 for all comparisons).

**Conclusions:**

Our findings suggest that participants who serve rural areas especially benefit from knowledge transfer within the TeleECHO community of practice. Additionally, the knowledge they receive comes from diverse information sources, emphasizing its multidisciplinary nature. Our results underscore the capacity of the TeleECHO model to leverage technology to promote rural health equity for miners.

## Introduction

Recent studies reveal an increasing prevalence and severity of pneumoconiosis (ie*,* dust-related lung diseases) among US coal workers since the late 1990s [1-7]. Data from the US Coal Workers Health Surveillance Program indicated that the 2017 prevalence of radiographic pneumoconiosis for coal miners with over 25 years of underground mining experience was greater than 10%, which was double the prevalence from the late 1990s. Similarly, the 2014 rate of complicated pneumoconiosis (a particularly deadly form) among long-tenured underground coal miners was 1.1%, compared to 0.3% at its lowest point in the late 1990s [7,8].

This re-emergence of pneumoconiosis presents unique challenges for rural communities. US counties with the highest mortality rates for pneumoconiosis are concentrated in rural contexts with long histories of mining, such as the Rocky Mountain states and central Appalachia [9]. The prevalence of radiographic pneumoconiosis and complicated pneumoconiosis in rural central Appalachian miners, in particular, is much higher than the national average [6]. While the number of miners requiring specialized care has increased, multidisciplinary expertise and access to complex care for pneumoconiosis have decreased in rural areas [10]. Compared with urban residents, residents of rural areas have less access to outpatient pulmonary rehabilitation [11] or pulmonologist services [12]. Rural practitioners also face unique challenges, including professional isolation and complex patient profiles [13], and describe multiple barriers to knowledge acquisition, such as resources and personal costs, physical distance, and time [14]. Such challenges amplify health inequities and mandate innovative approaches to enhance the health and well-being of rural miners, who constitute an underserved, geographically isolated, medically vulnerable, and often underinsured population [10].

Increasing access to education and mentoring of rural professionals involved in the multidisciplinary care of miners can ameliorate the current dearth of skilled expertise in mining-related diseases in rural areas. The multidisciplinary skills required include medicolegal, clinical, and “soft” skills, the latter including the interpersonal and communication skills needed to navigate highly collaborative work in the care of miners. Insufficient expertise among rural providers in these diverse skills demands innovative education and mentoring solutions.

The Miners’ Wellness TeleECHO (Extension for Community Health Outcomes) Program was established in 2016 to provide structured longitudinal multidisciplinary telementoring to members of professional groups caring for miners who reside in pneumoconiosis mortality hotspots in the United States [9]. Professional stakeholder groups include respiratory therapists, home health professionals, benefits counselors, lawyers or attorneys, clinicians, and others. These members from various stakeholder groups constitute a *digital community of practice*, or a group of people who “share a concern or a passion for something they do and learn how to do better as they interact regularly” [15]. This approach facilitates knowledge transfer and translation among participants. Knowledge transfer refers to the transmission of information and insights between people or groups [16,17], and knowledge translation involves enhancing users’ awareness of multidisciplinary knowledge and its use in day-to-day work and decision-making in the “real world” [16-18]. Importantly, little is known about how digital communities of practice transfer knowledge across professional stakeholder groups that tend to be geographically isolated, such as rural home health care workers and clinicians or specialists. Thus, examining the patterns of knowledge transfer in such communities of practice can provide insight into how technology can be leveraged to enhance care of complex disease in rural settings and how to promote shared objectives within communities of practice.

Preliminary studies indicate a favorable impact of the ECHO telementoring strategies on providers’ self-efficacy in the care of miners [19], adding to the knowledge base about how the ECHO model can enhance the management of other chronic diseases [20-22].

The rurality of the patient bases for those serving miners limits professionals’ capacity to seek and obtain specialized knowledge concerning the care of pneumoconiosis. This specialized knowledge tends to be concentrated within groups from urban areas, where academic health centers are located. Conversely, urban and suburban practitioners may have limited knowledge concerning the day-to-day challenges of rural patients undergoing treatment for complex diseases. The complexity and interdisciplinary nature of care for pneumoconiosis, coupled with the decline of multidisciplinary knowledge sources within rural areas, underscores the need for specialized knowledge transfer to underserved rural areas. Digital communities of practice are well-equipped to transfer multiple kinds of knowledge [23] across stakeholder groups. First, digital meetings help counteract large geographic distances, thereby providing opportunities for transmitting knowledge concerning facts (eg, know-what) and practical knowledge, skills, and expertise (eg, know-how) among otherwise isolated community members. Perhaps equally important, digital communities of practice help members make social connections and leverage their social networks to gain more access to practical skills and best practices and to adapt to the evolving needs of patients [23]. Understanding knowledge transfer between the community of practice participants from urban and rural patient or client bases is, therefore, essential but has not been fully evaluated—in turn, constituting a critical gap in knowledge. Addressing this knowledge gap can inform evidence-based interventions to enhance future efforts aimed at providing interdisciplinary care for rural miners.

This study evaluates the transfer of knowledge to professionals caring for miners using the digital community of practice approach. We integrate methods from social network analysis to examine patterns of knowledge transfer within and across stakeholder groups within a digital community of practice. We consider the association between the rurality of professionals’ patient or client base and (1) the number of knowledge sources from within the community of practice, (2) the number of knowledge sources outside one’s stakeholder group, (3) the number of knowledge sources within one’s stakeholder group, and (4) the range and heterogeneity in knowledge sources across stakeholder groups. Our study represents a crucial step in assessing the potential to reduce health inequity through greater investment in workforce diversity and interprofessional telementoring efforts that promote collaborative health care in medically underserved mining communities. Our study thus has important implications for understanding how technology fused with specialized expertise can be used to address complex health issues within rural, remote, and medically underserved communities and begin to address health inequities rooted in unequal access to medical care, more broadly. This approach may help rural communities counter the re-emergence of the pneumoconiosis epidemic.

## Methods

### Study Design

This is a cross-sectional study of professionals participating in the Miners’ Wellness TeleECHO Program, a community-university partnership between a small rural hospital—Miners’ Colfax Medical Center—and its academic partner—University of New Mexico School of Medicine, together constituting the “hub” site of experts. Stakeholder groups include clinical professional groups (clinicians, respiratory therapists, and home health professionals) and nonclinical professional groups (ie, benefits counselors, lawyers or attorneys, and others, including policymakers, administrators, and mine safety officers), constituting the “spoke” sites located across the United States. The hub and spoke partners regularly engage in telementoring and together form a digital community of practice.

### Recruitment

This study was based on a convenience sample of 70 participants who volunteered to complete this study’s survey, among all program attendees invited, during the 1-year study duration from September 12, 2018, to September 18, 2019. Core program faculty did not participate in the survey.

### Program Description

As detailed in a previous publication [19], TeleECHO sessions have a uniform format and are held at the same time twice every month, lasting 75 minutes. Program sessions begin with 10-minutes of introductions and announcements, followed by a 15-minute didactic delivered by an invited expert and a 20-minute facilitated question-answer session. Next, the program director facilitates a 30-minute interactive case discussion. Program sessions emphasize active learning through discussion, rather than didactic training. Participants earn continuing medical education (CME) credits without charge, upon completing a CME survey. A multidisciplinary curriculum committee follows a structured curriculum that is continually adapted based on the needs of the learning community, which are identified through review of the CME feedback reports. Attendance at ECHO sessions is open and voluntary, which allows those not presenting a case to view the didactic, partake in case discussions, contribute insight from professional experiences, and learn from the expert panel. Participants can also access experts at hub or spoke sites for urgent consultation outside of program sessions through telephone or electronic correspondence. Recorded didactic sessions are made available through a web-based archive.

### Program Development

Since July 2016, our program has used the ECHO model to provide long-term and structured telementoring in the care of miners. This approach deviates from traditional telemedicine where providers assume short-term direct care of individual patients [24]. Further, unlike webinars or traditional didactic lectures, the ECHO model provides an interactive discussion of cases with expert panels in real-time that is highly contextualized and adheres to key learning theory principles. As detailed in a previous publication [19], the ECHO model is based on the following five key principles. (1) The model uses internet-based technology for multipoint videoconferencing, to leverage scarce resources. (2) It uses an established disease-management model associated with best evidence for that disease that has been demonstrated to improve outcomes by reducing variation in processes of care and sharing best practices [21,22,25,26]. (3) It uses the principle of case-based learning for participants to learn with guidance from mentors, based on discussion, questions, and investigation of patient cases under their care. Over time, with iterative practice and feedback, participants gain knowledge and skills and progressively become more independent. (4) It creates a digital community of practice, which emphasizes reciprocity in the sharing of skills and information, and acknowledges that all participants bring useful expertise in the care of miners. Through regular interaction, community members increase their own expertise and that of other participants. As a result, the program aims to increase the ability of individual participants to (a) refer miners appropriately to other experts, (b) accept miner referrals from other experts, and (c) to serve as local experts for less experienced community professionals, thereby improving the care of miners. (5) Finally, it uses an internet-based database (ie, iECHO software) to monitor participant outcomes.

### Outcomes

We conceptualized knowledge transfer as the transmission of “facts, experiences, and insights” between people or groups [16,17]. We used a social network approach to examine knowledge transfer among community members by measuring respondents’ number of unique knowledge sources. We also considered the stakeholder group where knowledge originates, which allows us to examine the extent to which participants receive interdisciplinary knowledge from others outside of their own stakeholder groups as well as the overall distribution of knowledge sources across stakeholder groups.

We measured knowledge transfer sources by asking participants if they received new and important knowledge regarding the care of miners during and outside of TeleECHO sessions from each of the other participants. To measure knowledge transfer, respondents were given rosters that included names of all registered participants, with the option of providing additional names not on the roster. Rosters were arranged by stakeholder groups to reduce respondent burden and assist recall. We used these nominations to measure our dependent variables that capture unique dimensions of knowledge transfer. Our first dependent variable, *number of knowledge sources*, is the count of other participants from whom respondents received new and important knowledge (regardless of the source’s stakeholder group).

Apart from the number of knowledge sources, we tested whether rural participants report greater numbers of knowledge sources from outside of their primary stakeholder group. We thus measured the *number of cross-stakeholder knowledge sources*, which captures the number of participants from whom respondents received knowledge who were outside of respondents’ stakeholder group. We also analyzed the *number of same stakeholder knowledge sources*, with a measure capturing the number of participants from whom respondents received knowledge that were in the same stakeholder group as the focal respondent.

We also consider 2 dimensions of diversity in the sources of knowledge transfer among respondents. *Range* captures the extent to which individuals are connected to others from different social systems or interpersonal environments (eg, employers, associations, and schools) [27,28]. Importantly, a higher range level translates to greater access to nonredundant information [29]. We measure *knowledge source* *range* by calculating the number of unique stakeholder groups from which respondents reported receiving knowledge. This variable ranges from 0 to 5, with 0 indicating respondents reported no knowledge sources to 5 indicating respondents received knowledge from at least one member from each of the 5-stakeholder groups.

Our second measure capturing the diversity of knowledge sources is *heterogeneity of* *knowledge sources.* Our measure of heterogeneity of knowledge sources taps the distribution of knowledge sources across stakeholder groups for each respondent and is calculated as follows [30]:

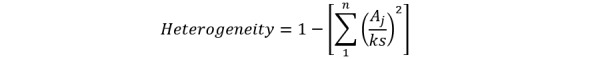



Here, *A_j_* is the number of knowledge sources that belong to a stakeholder group *j, ks* is the number of knowledge sources, and *n* is the total number of stakeholder groups from which the focal respondent reported receiving knowledge (ie, *knowledge source* *range*). Heterogeneity increases when respondents receive knowledge from a larger number of different stakeholder groups (ie, have high knowledge source range) and the knowledge sources are equally distributed across the stakeholder groups. In our study, this measure potentially ranges from 0 to 0.8, with higher values indicating greater heterogeneity in knowledge sources. Note, heterogeneity is undefined for respondents reporting 0 knowledge sources, which was the case for 2 of our respondents, who were excluded for analyses of heterogeneity.

### Independent Variables

Our focal independent variable captures the level of rurality among a respondent’s patient or client base. The measure is based on the percent of patients or clients who reside in rural areas, as self-reported by the participant. Initial responses were ordinal and included five categories: 1 (0% to 20%), 2 (21% to 40%), 3 (41% to 60%), 4 (61% to 80%), and 5 (81% to 100%). For this study, we collapsed the ordinal variable into a binary variable indicating rural patient or client base, which equals 1 if 41% to 100% of a respondent’s patient or client base resided in rural areas, and 0 if only 0% to 40% of their patients or clients lived in rural areas. We collapsed the categories for 2 reasons. First, exploratory analyses revealed that only 5 respondents reported serving a 21% to 40% rural patient or client base. Second, comparisons of the means of the knowledge source variables across levels of patient or client rurality suggested a threshold effect, with only minimal differences in the outcomes for those serving 0% to 20% versus 21% to 40% rural patient or client base but large differences between these combined categories and those serving a 41% or greater rural patient or client base. The results based on the original 5-category ordinal variable were similar to those presented in this study.

### Covariates

Multivariable models include control variables to account for potential confounding between the association between patient or client rurality and our outcomes. *Experienced versus fresh participant:* fresh participants were defined as those who first attended the community of practice in or after the summer of 2018 (defined as from May 9, 2018, onwards) versus experienced participants (defined by those who had first attended any time between July 1, 2016, and May 8, 2018). Experienced participants had greater cumulative participation and therefore, experience with the TeleECHO Program than fresh participants (11.4, SD 9.8 vs 4.6, SD 4.6 total sessions attended before or during this study’s timeframe; *P*=.03). This cutoff date was chosen based on the date of funding by the sponsor, which allowed the frequency of the TeleECHO Program to be raised from monthly to twice a month. Respondents’ *length of care for miners* taps the number of years each participant reported having served miners. Initial responses were measured in years. To aid in the interpretation of our regression results, we divide the reported number of years cared for miners by 10, so that the variable captures the number of decades respondents reported having cared for miners.

We also control for participant demographics. We control for age with 2 dummy variables indicating *51 to 60 years old* and *older than 60 years* (1=yes, 0=no) with *younger than 50 years old* serving as the reference category. *Male sex* is binary and indicates the respondent reported a male sex identity (1=yes, 0=no). Respondents reported their race and Hispanic ethnicity status. Based on the responses from these questions, respondents were initially categorized as either *Asian, non-Hispanic-Black or African American*, *Hispanic, multiracial or some other race,* or *non-Hispanic White.* We report the percentages of respondents in each race or ethnic category but collapsed categories into a binary variable indicating *non-White* (1=yes, non-Hispanic White is the reference) in our regression analyses due to the small sizes of non-White racial or ethnic groups in the sample. Alternative methods of collapsing race or ethnic categories resulted in similar findings as those presented here.

### Data Collection

The program monitored the number of sessions, learners, unique learners, geographical sites of learners, and patient cases presented (using the iECHO software). Survey data were collected using the REDCap (Research Electronic Data Capture; Vanderbilt University), a secure web app for building and managing online surveys and databases.

### Analytic Strategy

All analyses were conducted in Stata/MP (version 16.0; StataCorp LLC). We used negative binomial regressions to analyze the total number of knowledge sources, number of cross-stakeholder knowledge sources, and number of same stakeholder knowledge sources, which were discrete counts and were over dispersed. Ordinary least squares regression was used to analyze knowledge source diversity and knowledge source heterogeneity. Model coefficients (*b*) and SE were used to summarize effect sizes. Data missingness due to nonresponse was minimal, with 2 respondents declining to report their age, 1 respondent declining to report on length of care for miners, and 1 respondent declining to report on rurality (for a total of 3 respondents having missing data on at least 1 variable). Missing values on these measures were imputed using the Stata *ice* procedure [31], and models were estimated with 10 imputed data sets using the *mi* command suite in Stata 16. The results based on unimputed data using listwise deletion were nearly identical to those presented here.

### Ethics Considerations

Approval was obtained from the institutional review board, Human Research Protections Office, at the University of New Mexico Health Sciences Center (18-386). Anonymized consent was obtained from all participants. Study data were deidentified for analysis to maintain confidentiality. All participants were provided an electronic merchandise card of US $50 upon survey completion.

## Results

Table 1 shows the descriptive characteristics of the 70 ECHO participants caring for pneumoconiosis in a cross-sectional study during the timeframe of 2018-2019.

**Table 1:** Descriptive characteristics of study participants (N=70).

Characteristics		Value
**Knowledge source variables, mean (SD)**		
	Number of knowledge sources (N=70)	4.46 (3.16)
	Same stakeholder knowledge sources (n=61)	1.72 (1.46)
	Cross-stakeholder knowledge sources (n=61)	2.80 (2.63)
	Knowledge source range (N=70)	2.50 (1.29)
	Heterogeneity of knowledge sources (n=68)	0.44 (0.30)
**Age group (y), n (%)**		
	50 or younger	36 (51)
	51 to 60	15 (21)
	Older than 60	19 (27)
**Sex, n (%)**		
	Female	45 (64)
	Male	25 (36)
**Race or ethnicity, n (%)**		
	Asian	6 (9)
	Hispanic	5 (7)
	Non-Hispanic Black or African American	1 (1)
	Non-Hispanic White	55 (79)
	Other	3 (4)
**Primary stakeholder group, n (%)**		
	Clinician	20 (29)
	Respiratory therapist	12 (17)
	Lawyer or attorney	7 (10)
	Benefits counselor	8 (11)
	Home health professional	14 (20)
	Others	9 (13)
**Rurality of patient or client base, n (%)**		
	Nonrural patient or client base	19 (27)
	Rural patient or client base	51 (73)
**Participant experience, n (%)**		
	Fresh participant	40 (57)
	Experienced participant	30 (43)
Decades serving miners (N=70), mean (SD)		0.76 (0.72)

### Knowledge Source Variables Among all Participants

Respondents, on average, identified 4.46 (SD 3.16) unique knowledge sources within the community. Respondents, on average, reported greater numbers of cross-stakeholder knowledge sources (2.80, SD 2.63) than same stakeholder knowledge sources (1.72, SD 1.46). The mean knowledge source range was 2.50 (SD 1.29), indicating that, on average, respondents received knowledge sources from roughly half of the 5 stakeholder groups. Finally, the mean heterogeneity of knowledge sources, which can range between 0 and 0.80, was near the midpoint of the scale at 0.44 (SD 0.30).

### Knowledge Source Variables Among Participants Serving Rural Versus Nonrural Bases

We explain the means of the knowledge source measures among those serving rural versus nonrural patient or client bases. Those serving rural patient or client bases, on average, reported 5.00 (SD 3.13) unique knowledge sources compared to 3.00 (SD 2.88) among those primarily serving nonrural patients or clients. There was only a minor difference in the mean number of same stakeholder knowledge sources for those serving rural (1.68, SD 1.42) versus nonrural (1.86, SD 1.66) patients or clients. However, rural providers, on average, identified 3.30 (SD 2.61) cross-stakeholder knowledge sources, whereas nonrural providers, on average, identified 1.14 (SD 1.99) cross-stakeholder knowledge sources. Finally, comparing the measures of diversity of knowledge sources, those serving rural patients or clients had a higher mean knowledge source range 2.78 (SD 1.22) versus 1.74 (SD 1.19) and mean knowledge source heterogeneity 0.52 (SD 0.25) versus 0.23 (SD 0.31) than those serving primarily nonrural patients or clients.

### Multivariable Results

Table 2 presents results from multivariable regression models of the different dimensions of knowledge transfer.

**Table 2:** Multivariable regression analyses of knowledge transfer among digital community of practice members caring for pneumoconiosis in a cross-sectional study during the timeframe of 2018-2019^a^,^b^.

Independent variables		Model 1: number of knowledge sources (N=70)			Model 2: same stakeholder knowledge sources (n=61)			Model 3: cross-stakeholder knowledge sources (n=61)			Model 4: knowledge source range (n=70)			Model 5: heterogeneity of knowledge, sources (n=68)	
		b (SE)	*P* value	b (SE)		*P* value	b (SE)		*P* value	b (SE)		*P* value	b (SE)		*P* value
											
Rurality		0.50 (0.22)	.02	0.04 (0.26)		.89	0.91 (0.37)		.01	0.92 (0.36)		.01	0.25 (0.08)		.003
**Age (y; ≤50 y is the reference)**															
	51-60	–0.05 (0.24)	.84	0.67 (0.30)		.03	–0.33 (0.34)		.34	–0.06 (0.41)		.88	–0.06 (0.09)		.53
	>60	–0.35 (0.28)	.22	0.14 (0.34)		.68	–0.52 (0.40)		.19	–0.79 (0.44)		.08	–0.16 (0.10)		.14
Male sex (female sex is the reference)		–0.11 (0.19)	.57	–0.34 (0.27)		.21	–0.07 (0.27)		.79	–0.39 (0.34)		.25	–0.08 (0.08)		.30
Non-White (non-Hispanic White is the reference)		0.32 (0.22)	.14	–0.24 (0.30)		.41	0.62 (0.34)		.07	0.38 (0.37)		.32	0.11 (0.09)		.19
**Stakeholder group (clinical provider is the reference)**															
	Respiratory therapist	0.15 (0.27)	.58	–0.50 (0.36)		.17	0.54 (0.37)		.15	0.37 (0.48)		.44	0.17 (0.11)		.12
	Lawyer or attorney	–0.04 (0.32)	.89	–0.50 (0.41)		.23	0.36 (0.45)		.43	–0.34 (0.55)		.55	0.01 (0.12)		.95
	Benefits counselor	0.29 (0.29)	.31	–0.82 (0.43)		.06	0.84 (0.39)		.03	0.67 (0.51)		.20	0.18 (0.12)		.13
	Home health professional	0.34 (0.25)	.18	0.20 (0.30)		.50	0.38 (0.36)		.29	–0.17 (0.45)		.72	–0.01 (0.10)		.93
	Others	0.20 (0.29)	.50	—^c^		—	—		—	0.78 (0.50)		.13	0.21 (0.12)		.08
Experienced participant (fresh participant is the reference)		0.37 (0.17)	.03	–0.17 (0.21)		.42	0.74 (0.25)		.003	0.70 (0.31)		.03	0.12 (0.07)		.10
Decades serving miners		0.18 (0.16)	.24	0.08 (0.19)		.68	0.17 (0.24)		.47	0.46 (0.26)		.08	0.11 (0.06)		.08
Intercept		0.68 (0.30)	.02	0.66 (0.35)		.06	–0.58 (0.45)		.20	10.28 (0.49)		.01	0.11 (0.11)		.33

^a^*P* values obtained from 2-tailed tests.

^b^In these models, our dependent variables are knowledge sources, that is, other participants from whom respondents (ie, targets) received new and important knowledge (within and outside the source’s stakeholder group).

^c^Not available.

### Number of Knowledge Sources

Model 1 examines the number of knowledge sources using a negative binomial regression. The results indicate that rural patient or client base is positively associated with the number of knowledge sources (*b*=0.50; *P*=.02). This finding suggests that providers serving rural clients or patients identify greater numbers of knowledge sources within the community of practice than participants whose patients or clients reside in nonrural areas, even after accounting for key confounders. Compared with fresh participants, experienced participants report greater numbers of knowledge sources (*b=*0.37; *P*=.03). No other participant characteristics were significantly associated with the number of knowledge sources (all *P*>.05).

### Number of Same Stakeholder and Cross-Stakeholder Knowledge Sources

Models 2 and 3 examine the number of same stakeholder and cross-stakeholder knowledge sources, respectively. These models provide insight into whether participants tend to identify knowledge sources from within or outside of their primary stakeholder groups. Note, participants from “Other” stakeholder groups were dropped from Models 2 and 3, as they by definition have all different stakeholder ties and 0 same stakeholder ties. The results from Model 2 indicate that rural patient or client base has a nonsignificant association with the number of same stakeholder knowledge sources (*b*=0.04; *P*=.89). Compared with those aged 50 years or younger, participants between the ages of 51 and 60 years report more ties to members of the same stakeholder group (*b*=0.67; *P*=.03). Turning to Model 3, which examines the number of cross-stakeholder knowledge sources, the rurality of the patient or client base was positively associated with the number of cross-stakeholder ties (*b=*0*.*91; *P*=*.*01). This indicates that participants serving larger proportions of rural patients or clients reported larger numbers of cross-stakeholder knowledge sources than those serving smaller proportions of rural patients or clients. Additionally, experienced participants report larger numbers of cross-stakeholder knowledge sources than fresh participants (*b*=0.74; *P*=.003) and benefits counselors report larger numbers of cross-stakeholder knowledge sources than clinicians (*b*=0.84; *P*=.03).

### Range and Heterogeneity of Knowledge Sources

The final models in Table 2 examine the range and heterogeneity of participants’ knowledge sources. These models provide insight into the number of different stakeholder groups from which participants received knowledge, and the extent to which participants’ knowledge sources are equally dispersed across different stakeholder groups. Turning to Model 4, which is a linear regression of knowledge source range, we found that a rural patient or client base has a positive and significant coefficient (*b*=0.92; *P*=.01). Model 4 also indicates that experienced participants reported higher knowledge source range than fresh and new participants (*b*=0.70; *P*=.03).

Finally, Model 5 examines the association between rurality and participants’ knowledge source heterogeneity. Whereas range is the count of the number of unique stakeholder groups from which participants receive knowledge, knowledge source heterogeneity also assesses whether stakeholders from which participants receive knowledge tend to be concentrated in 1 stakeholder group (low heterogeneity) versus equally distributed across multiple groups (high heterogeneity). Note, that because knowledge source heterogeneity can only be measured among participants with at least one knowledge source, Model 5 excludes 2 respondents who reported 0 knowledge sources. Patient or client rurality was positively associated with participants’ heterogeneity of knowledge sources (*b*=0.25; *P*=.003), indicating knowledge sources are more equally distributed across stakeholder groups as the rurality of their patient and client bases increased.

## Discussion

### Principal Findings

Community of practice participants with higher proportions of rural patient or client base, on average, report more knowledge sources overall, more knowledge sources from outside of their stakeholder groups, a higher knowledge source range, and greater heterogeneity of knowledge sources than those with a lower proportion of rural patient or client base. These findings were confirmed after adjustment for potential confounders in regression analyses. More broadly, these findings suggest participants who serve rural areas especially benefit from knowledge transfer within the TeleECHO community of practice. Additionally, the knowledge they receive comes from diverse information sources, emphasizing its multidisciplinary nature.

Further, 1 primary objective of Project ECHO is to decentralize knowledge for the care of patients through exchanging insights and information. Knowledge transfer is key to enhancing the care of complex disease by timely, evidence-based information shared by experts who have used, amplified, and applied this knowledge with interested professionals who (1) are seeking knowledge to assist their patients or clients and (2) through its application, increase access to complex disease care for patients in rural and underserved communities. Project ECHO supports knowledge transfer within the community of practice, through experts sharing and discussing evidence in association with challenging questions with which professionals at program spoke sites are wrestling. Our study suggests this knowledge transfer may be particularly effective among professionals with longer experience with the program.

Professionals in rural mining communities often lack access to traditional knowledge sources. This disparity results from professional isolation; challenges with continuing professional education that requires travel to a distant site for participation with resultant closure of their practices, often without adequate coverage available; and unavailability of specialists with more in-depth knowledge about the clinical, medicolegal, and interpersonal aspects of care of miners. The need to increase access to information for rural professionals is, therefore, obvious. To this end, information technology has come to the fore. However, research suggests that even when electronic information services are provided to rural practitioners, they may not be well used [32]. The lack of information handling skills, lack of time, and perceived peripherality to the job are all seen as major constraints [33,34]. However, our study challenges this belief by demonstrating that professionals serving rural areas especially benefit from access to knowledge through the innovative TeleECHO model, which would otherwise remain siloed within stakeholder groups. Further, the knowledge source range and heterogeneity that the TeleECHO model promotes may allow greater access to thought-provoking ideas that foster learning and other growth-enhancing actions [27,35]. To the best of our knowledge, our approach of studying patterns of knowledge transfer, using social network analysis tools, has never been used previously.

### Strengths

Our study has multiple strengths. It involves an innovative intervention that addresses the barriers to the care of miners by using the TeleECHO model, which provides a multidisciplinary community of practice approach, using internet-based technology, an approach that has been well studied in other diseases [21,22,25,26]. This study is topical and significant because it addresses a critical gap related to the emerging pneumoconiosis epidemic in the rural United States. Since the ECHO model has been adopted nationally and globally to improve rural access to care for patients with numerous chronic diseases, there already exists infrastructure to allow for rapid scaling of the Miners’ Wellness TeleECHO Program nationally and globally.

### Limitations

There are also limitations to this study. We are unable to correlate knowledge transfer to patient outcomes or changes in provider behavior. We have, however, previously published a listing of qualitative changes that our ECHO participants reported they were going to make in their practice, obtained as part of a CME survey requested at the end of each TeleECHO session [36]. Although a small sample size raises the possibility of a type I error, individual professionals and teams of professionals trained in the ECHO model can reach a large number of miners, with the potential for creating exponential change. High-risk individuals who did not volunteer to participate in this study would not have provided information in the estimation of the program effects, thus introducing an element of potential participation bias. The knowledge transfer instrument was not validated in this study. Program participants had variable competencies, with varying levels of sophistication, commitment, expertise, experience, and historic levels of collaboration within the TeleECHO Program. However, adjustment for participant experience with the TeleECHO Program or length of care for miners in the multivariable models did not change our study findings. Intergenerational, interinstitutional, and rural-urban disparities in ability to leverage technology by participating professionals may challenge empirical examinations of knowledge transfer. Finally, data limitations, including survey nonparticipation by the core program faculty and survey nonresponse among the TeleECHO Program participants, preclude the use of complex social network analysis methods (eg, exponential random graph models) commonly used to examine network selection processes in our study. Although our methods are adequate for examining associations between participating characteristics and the number, range, and heterogeneity of knowledge sources, we are unable to examine how network processes such as reciprocal knowledge transfer operate within the learning community.

### Conclusions

Despite these limitations, our findings suggest participants who serve rural areas especially benefit from knowledge transfer within the TeleECHO community of practice. Additionally, the knowledge they receive comes from diverse information sources, emphasizing its multidisciplinary nature. Our results underscore the capacity of the Project ECHO model to leverage technology and workforce diversity to facilitate knowledge transfer to rural professionals and ultimately promote health equity among rural and medically underserved mining communities. Although this approach addresses a critical gap related to the emerging pneumoconiosis epidemic in rural United States, future research will evaluate whether this translates into improved patient outcomes in rural mining communities.

## Abbreviations

CME: continuing medical education
ECHO: Extension for Community Health Outcomes
REDCap: Research Electronic Data Capture

## Data Availability

The data sets generated or analyzed during this study are available from the corresponding author on reasonable request.

## References

[1] Petsonk EL, Rose C, Cohen R (2013). Coal mine dust lung disease. new lessons from old exposure. Am J Respir Crit Care Med.

[2] Laney AS, Blackley DJ, Halldin CN (2017). Radiographic disease progression in contemporary US coal miners with progressive massive fibrosis. Occup Environ Med.

[3] Blackley DJ, Halldin CN, Wang ML, Laney AS (2014). Small mine size is associated with lung function abnormality and pneumoconiosis among underground coal miners in Kentucky, Virginia and West Virginia. Occup Environ Med.

[4] Blackley DJ, Reynolds LE, Short C, Carson R, Storey E, Halldin CN, Laney AS (2018). Progressive massive fibrosis in coal miners from 3 clinics in Virginia. JAMA.

[5] Antao VCDS, Petsonk EL, Sokolow LZ, Wolfe AL, Pinheiro GA, Hale JM, Attfield MD (2005). Rapidly progressive coal workers' pneumoconiosis in the United States: geographic clustering and other factors. Occup Environ Med.

[6] Blackley DJ, Halldin CN, Laney AS (2018). Continued increase in prevalence of coal workers' pneumoconiosis in the United States, 1970-2017. Am J Public Health.

[7] Laney AS, Attfield MD (2010). Coal workers' pneumoconiosis and progressive massive fibrosis are increasingly more prevalent among workers in small underground coal mines in the United States. Occup Environ Med.

[8] Blackley DJ, Halldin CN, Laney AS (2014). Resurgence of a debilitating and entirely preventable respiratory disease among working coal miners. Am J Respir Crit Care Med.

[9] Dwyer-Lindgren L, Bertozzi-Villa A, Stubbs RW, Morozoff C, Shirude S, Naghavi M, Mokdad AH, Murray CJL (2017). Trends and patterns of differences in chronic respiratory disease mortality among US counties, 1980-2014. JAMA.

[10] Evans K, Lerch S, Boyce TW, Myers OB, Kocher E, Cook LS, Sood A (2016). An innovative approach to enhancing access to medical screening for miners using a mobile clinic with telemedicine capability. J Health Care Poor Underserved.

[11] Fan VS, Giardino ND, Blough DK, Kaplan RM, Ramsey SD, Nett Research Group (2008). Costs of pulmonary rehabilitation and predictors of adherence in the national emphysema treatment trial. COPD.

[12] Croft JB, Lu H, Zhang X, Holt JB (2016). Geographic accessibility of pulmonologists for adults with COPD: United States, 2013. Chest.

[13] Miedema B, Hamilton R, Fortin P, Easley J, Tatemichi S (2009). The challenges and rewards of rural family practice in New Brunswick, Canada: lessons for retention. Rural Remote Health.

[14] Crockett LK, Leggett C, Curran JA, Knisley L, Brockman G, Scott SD, Hartling L, Jabbour M, Klassen TP (2018). Knowledge sharing between general and pediatric emergency departments: connections, barriers, and opportunities. CJEM.

[15] Wenger E (1998). Communities of Practice: Learning, Meaning and Identity.

[16] Hargadon A, Sutton RI (1997). Technology brokering and innovation in a product development firm. Adm Sci Q.

[17] Tasselli S (2015). Social networks and inter-professional knowledge transfer: the case of healthcare professionals. Organ Stud.

[18] Gallagher DM, Hirschhorn LR, Lorenz LS, Piya P (2017). Developing a community of practice for HIV care: supporting knowledge translation in a regional training initiative. J Contin Educ Health Prof.

[19] Sood A, Assad N, Jarrell W, Kalishman S, Le Suer K, Murillo S, Myers O, Rochelle R, Salveson S, Soller B, Walker J, Wissore B, Pollard C (2020). A virtual community-of-practice approach by rural stakeholders in managing pneumoconiosis in the USA: a cross-sectional analysis. Rural Remote Health.

[20] Zhou C, Crawford A, Serhal E, Kurdyak P, Sockalingam S (2016). The impact of project ECHO on participant and patient outcomes: a systematic review. Acad Med.

[21] Bouchonville MF, Hager BW, Kirk JB, Qualls CR, Arora S (2018). Endo ECHO improves primary care provider and community health worker self-efficacy in complex diabetes management in medically underserved communities. Endocr Pract.

[22] Arora S, Kalishman S, Thornton K, Dion D, Murata G, Deming P, Parish B, Brown J, Komaromy M, Colleran K, Bankhurst A, Katzman J, Harkins M, Curet L, Cosgrove E, Pak W (2010). Expanding access to hepatitis C virus treatment--Extension for Community Healthcare Outcomes (ECHO) project: disruptive innovation in specialty care. Hepatology.

[23] Lundvall B, Johnson B (1994). The learning economy. J Ind Stu.

[24] Ruvuna L, Sood A (2020). Epidemiology of chronic obstructive pulmonary disease. Clin Chest Med.

[25] Deming P, Arora S (2011). Taribavirin in the treatment of hepatitis C. Expert Opin Investig Drugs.

[26] Arora S, Thornton K, Murata G, Deming P, Kalishman S, Dion D, Parish B, Burke T, Pak W, Dunkelberg J, Kistin M, Brown J, Jenkusky S, Komaromy M, Qualls C (2011). Outcomes of treatment for hepatitis C virus infection by primary care providers. N Engl J Med.

[27] Higgins MC, Kram KE (2001). Reconceptualizing mentoring at work: a developmental network perspective. Acad Manage Rev.

[28] Haines VA, Hurlbert JS (1992). Network range and health. J Health Soc Behav.

[29] Granovetter MS (1973). The strength of weak ties. Am J Sociol.

[30] (2001). National Longitudinal Study of Adolescent Health. Network Variables Codebook.

[31] Royston P (2004). Multiple imputation of missing values. Stata J.

[32] Asthana S, Halliday J (2004). What can rural agencies do to address the additional costs of rural services? a typology of rural service innovation. Health Soc Care Community.

[33] Farmer J, Williams D (2000). Effective rural health information services. Health Libr Rev.

[34] Farmer J, Richardson A (1997). Information for trained nurses in remote areas: do electronically networked resources provide an answer?. Health Libr Rev.

[35] Shen Y, Kram KE (2011). Expatriates' developmental networks: network diversity, base, and support functions. Career Dev Int.

[36] Sood A, Pollard C, Kalishman S, Assad N, LeSuer K, Khattar R, Soller B, Myers O (2020). Telementoring of healthcare teams in the care of miners. ATS Sch.

